# Supplementary material for: A pathogenic human Orai1 mutation unmasks STIM1-independent rapid inactivation of Orai1 channels
Source: eLife. 2023 Feb 20;12:e82281. doi: 10.7554/eLife.82281 (PMC9991058; doi:10.7554/eLife.82281)
Supplement: Figure 1—source data 1. [file elife-82281-fig1-data1.docx]

Figure 1 – Source Data. Mutational analysis and contact dots analysis of Orai1 L138

**Figure 1C**

| **TM2 Contact Analysis** | | | | |
| --- | --- | --- | --- | --- |
| **dOrai Residue** | **hOrai1 Residue** | Total Contacts | Contacts to TM1 | % of Contacts with TM1 |
| **M191** | **L119** | 0 | 0 | * |
| **L192** | **L120** | 40 | 0 | * |
| **I193** | **I121** | 58 | 0 | * |
| **A194** | **A122** | 0 | 0 | * |
| **F195** | **F123** | 235 | 1 | 0% |
| **A196** | **S124** | 108 | 0 | * |
| **I197** | **A125** | 44 | 0 | * |
| **C198** | **C126** | 182 | 0 | * |
| **T199** | **T127** | 261 | 102 | 39% |
| **T200** | **T128** | 191 | 0 | * |
| **L201** | **V129** | 126 | 0 | * |
| **L202** | **L130** | 288 | 52 | 18% |
| **V203** | **V131** | 155 | 3 | 2% |
| **A204** | **A132** | 176 | 0 | * |
| **V205** | **V133** | 225 | 0 | * |
| **H206** | **H134** | 205 | 125 | 61% |
| **M207** | **L135** | 257 | 63 | 25% |
| **L208** | **F136** | 90 | 0 | * |
| **A209** | **A137** | 26 | 0 | * |
| **L210** | **L138** | 215 | 133 | 62% |
| **M211** | **M139** | 195 | 0 | * |
| **I212** | **I140** | 163 | 0 | * |
| **S213** | **S141** | 30 | 30 | 100% |
| **T214** | **T142** | 14 | 0 | * |
| **C215** | **C143** | 0 | 0 | * |
| **I216** | **I144** | 64 | 0 | * |
| **L217** | **L145** | 0 | 0 | * |
| **P218** | **P146** | 0 | 0 | * |
| **N219** | **N147** | 7 | 0 | * |

*not calculated due to zero contacts made with TM1.

**Figure 1D-E**

| **Orai1 alone** | | | | |
| --- | --- | --- | --- | --- |
| Mutant | Current Density (pA/pF ± SEM) | N | T-test p-value  (versus WT) | Reversal Potential (mV ± SEM) |
| **WT** | -0.2 ± 0.03 | 5 | N/A | * |
| **L138A** | -0.1 ± 0.07 | 4 | 0.59 | * |
| **L138C** | -0.2 ± 0.05 | 5 | 0.97 | * |
| **L138D** | -0.3 ± 0.1 | 5 | 0.27 | * |
| **L138E** | -0.1 ± 0.02 | 5 | 0.060 | * |
| **L138F** | -2.9 ± 0.8 | 6 | 0.016 | 53.5 ± 2.5 |
| **L138G** | -0.2 ± 0.05 | 5 | 0.54 | * |
| **L138H** | -0.2 ± 0.08 | 5 | 0.82 | * |
| **L138I** | -1.5 ± 0.3 | 5 | 0.015 | * |
| **L138K** | -0.4 ± 0.08 | 5 | 0.064 | * |
| **L138M** | -0.4 ± 0.2 | 5 | 0.29 | * |
| **L138N** | -0.1 ± 0.03 | 6 | 0.45 | * |
| **L138P** | -0.3 ± 0.05 | 5 | 0.070 | * |
| **L138Q** | -0.2 ± 0.06 | 4 | 0.47 | * |
| **L138R** | -0.1 ± 0.02 | 5 | 0.075 | * |
| **L138S** | -0.1 ± 0.03 | 6 | 0.24 | * |
| **L138T** | -0.2 ± 0.1 | 5 | 0.72 | * |
| **L138V** | -0.1 ± 0.03 | 6 | 0.51 | * |
| **L138W** | -0.4 ± 0.1 | 5 | 0.14 | * |
| **L138Y** | -4.8 ± 1.0 | 5 | 0.0090 | 54.3 ± 6.3 |

*not measured due to small current amplitudes (<2 pA/pF).

**Figure 1 – figure supplement 1A-B**

| **Orai1 with STIM1** | | | | |
| --- | --- | --- | --- | --- |
| Mutant | Current Density  (pA/pF ± SEM) | N | T-test p-value (versus WT) | Reversal Potential (mV ± SEM) |
| **WT** | -29.1 ± 8.6 | 5 | N/A | 52.0 ± 3.9 |
| **L138A** | -0.69 ± 0.3 | 5 | 0.030 | * |
| **L138C** | -1.5 ± 0.3 | 5 | 0.033 | * |
| **L138D** | -0.5 ± 0.1 | 4 | 0.029 | * |
| **L138E** | -1.2 ± 0.3 | 5 | 0.032 | * |
| **L138F** | -38.4 ± 8.9 | 5 | 0.47 | 62.6 ± 3.2 |
| **L138G** | -0.33 ± 0.1 | 6 | 0.029 | * |
| **L138H** | -2.7 ± 0.7 | 5 | 0.037 | 54.0 ± 7.1 |
| **L138I** | -18.6 ± 5.1 | 5 | 0.33 | 42.6 ± 5.6 |
| **L138K** | -3.8 ± 1.3 | 5 | 0.042 | 48.3 ± 12.0 |
| **L138M** | -34.9 ± 11.0 | 5 | 0.69 | 58.2 ± 4.9 |
| **L138N** | -0.9 ± 0.2 | 5 | 0.030 | * |
| **L138P** | -1.7 ± 0.8 | 5 | 0.033 | * |
| **L138Q** | -2.4 ± 0.6 | 6 | 0.036 | 52.7 ± 10.4 |
| **L138R** | -1.9 ± 0.7 | 7 | 0.034 | * |
| **L138S** | -0.7 ± 0.2 | 5 | 0.030 | * |
| **L138T** | -3.2 ± 0.5 | 5 | 0.039 | 48.6 ± 5.8 |
| **L138V** | -26.4 ± 6.6 | 6 | 0.81 | 47.2 ± 3.0 |
| **L138W** | -20.4 ± 1.6 | 4 | 0.37 | 61.3 ± 5.7 |
| **L138Y** | -25.4 ± 4.3 | 7 | 0.71 | 57.0 ± 3.6 |

*not measured due to small current amplitudes (<2 pA/pF).

**Figure 1 – figure supplement 1E**

| **Orai1-YFP with CFP-CAD** | | | |
| --- | --- | --- | --- |
| Mutant | E-FRET ± SEM | N | T-test p-value  (versus WT) |
| **WT** | 0.304 ± 0.0066 | 51 | N/A |
| **L138A** | 0.304 ± 0.0068 | 57 | 0.98 |
