# Supplementary material for: A pathogenic human Orai1 mutation unmasks STIM1-independent rapid inactivation of Orai1 channels
Source: eLife. 2023 Feb 20;12:e82281. doi: 10.7554/eLife.82281 (PMC9991058; doi:10.7554/eLife.82281)
Supplement: Figure 2—source data 1. [file elife-82281-fig2-data1.docx]

Figure 2 – Source Data. Double mutants of L138 with neighboring TM1 residues reveals an interaction with T92.

**Figure 2B**

| **Orai1 alone** | | | |
| --- | --- | --- | --- |
| Mutant | Current Density (pA/pF ± SEM) | N | T-test p-value  (versus L138F) |
| **WT** | -0.2 ± 0.03 | 5 | 0.016 |
| **L138F** | -2.9 ± 0.8 | 6 | N/A |
| **A94G/L138F** | -3.6 ± 0.9 | 5 | 0.52 |
| **S93G/L138F** | -0.5 ± 0.1 | 6 | 0.027 |
| **T92G/L138F** | -0.2 ± 0.1 | 6 | 0.017 |

**Figure 2D**

| **Orai1 alone** | | | |
| --- | --- | --- | --- |
| Mutant | Current Density (pA/pF ± SEM) | N | T-test p-value  (versus WT) |
| **WT** | -0.2 ± 0.03 | 5 | N/A |
| **A94F** | -0.1 ± 0.02 | 5 | 0.019 |
| **S93F** | -0.6 ± 0.2 | 5 | 0.15 |
| **T92F** | -33.1 ± 2.8 | 5 | 3.0*10^-4^ |
| **T92F/L138G** | -0.5 ± 0.2 | 5 | 0.17 |

**Figure 2F**

| **Orai1 with STIM1** | | | |
| --- | --- | --- | --- |
| Mutant | Current Density (pA/pF ± SEM) | N | T-test p-value  (versus L138G) |
| **WT** | -29.1 ± 8.6 | 5 | 0.029 |
| **L138G** | -0.33 ± 0.1 | 6 | N/A |
| **T92F/L138G** | -30.7 ± 6.5 | 6 | 5.4*10^-3^ |

**Figure 2 – figure supplement 1A**

| **T92G/L138F** | | | |
| --- | --- | --- | --- |
| Mutant | Current Density (pA/pF ± SEM) | N | T-test p-value  (versus no STIM1) |
| **No STIM1** | -0.2 ± 0.1 | 6 | N/A |
| **With STIM1** | -20.2 ± 9.8 | 3 | 0.18 |

| **Orai1 alone** | | | |
| --- | --- | --- | --- |
| Mutant | Current Density (pA/pF ± SEM) | N | T-test p-value  (versus WT) |
| **WT** | -29.1 ± 8.6 | 5 | N/A |
| **S93F** | -3.6 ± 1.6 | 5 | 0.040 |
| **A94F** | -0.8 ± 0.6 | 5 | 0.030 |

**Figure 2 – figure supplement 1B**
