# Supplementary material for: A pathogenic human Orai1 mutation unmasks STIM1-independent rapid inactivation of Orai1 channels
Source: eLife. 2023 Feb 20;12:e82281. doi: 10.7554/eLife.82281 (PMC9991058; doi:10.7554/eLife.82281)
Supplement: Figure 3—source data 1. [file elife-82281-fig3-data1.docx]

Figure 3 – Source Data. Orai1 channel activity is correlated with side chain size at T92.

**Figure 3A-B**

| **Orai1 alone** | | | | |
| --- | --- | --- | --- | --- |
| Mutant | Current Density (pA/pF ± SEM) | N | T-test p-value  (versus WT) | Reversal Potential (mV ± SEM) |
| **WT** | -0.2 ± 0.03 | 5 | N/A | * |
| **T92A** | -0.4 ± 0.1 | 5 | 0.088 | * |
| **T92C** | -0.5 ± 0.2 | 5 | 0.19 | * |
| **T92D** | -0.2 ± 0.03 | 3 | 0.13 | * |
| **T92E** | -0.1 ± 0.04 | 5 | 0.33 | * |
| **T92F** | -33.1 ± 2.8 | 5 | 3.0*10^-4^ | 44.0 ± 5.1 |
| **T92G** | -0.1 ± 0.03 | 5 | 0.17 | * |
| **T92H** | -8.4 ± 2.0 | 5 | 0.016 | 42.3 ± 7.9 |
| **T92I** | -9.1 ± 2.0 | 4 | 0.022 | 40.2 ± 5.7 |
| **T92K** | -1.8 ± 0.7 | 5 | 0.082 | * |
| **T92L** | -29.3 ± 5.0 | 5 | 4.5*10^-3^ | 55.6 ± 10.1 |
| **T92M** | -35.0 ± 6.0 | 5 | 4.4*10^-3^ | 45.6 ± 4.3 |
| **T92N** | -0.5 ± 0.3 | 4 | 0.24 | * |
| **T92P** | -0.6 ± 0.1 | 5 | 0.047 | * |
| **T92Q** | -0.3 ± 0.1 | 4 | 0.28 | * |
| **T92R** | -0.8 ± 0.2 | 5 | 0.21 | * |
| **T92S** | -0.3 ± 0.09 | 5 | 0.19 | * |
| **T92V** | -6.7 ± 2.4 | 6 | 0.042 | 50.3 ± 4.7 |
| **T92W** | -27.7 ± 3.8 | 8 | 1.7*10^-4^ | 53.5 ± 2.8 |
| **T92Y** | -35.0 ± 6.2 | 6 | 2.4*10^-3^ | 48.5 ± 6.8 |

*not measured due to small current amplitudes (<2 pA/pF).

**Figure 3 – figure supplement 1A-B**

| **Orai1 with STIM1** | | | | |
| --- | --- | --- | --- | --- |
| Mutant | Current Density  (pA/pF ± SEM) | N | T-test p-value  (versus WT) | Reversal Potential (mV ± SEM) |
| **WT** | -29.1 ± 8.6 | 5 | N/A | 52.0 ± 3.9 |
| **T92A** | -38.9 ± 7.9 | 5 | 0.43 | 59.8 ± 5.6 |
| **T92C** | -22.9 ± 4.8 | 5 | 0.55 | 55.0 ± 2.6 |
| **T92D** | -0.6 ± 0.2 | 5 | 0.030 | * |
| **T92E** | -14.1 ± 4.6 | 4 | 0.17 | 45.0 ± 11.1 |
| **T92F** | -21.1 ± 4.2 | 4 | 0.43 | 56.0 ± 9.0 |
| **T92G** | -7.7 ± 1.0 | 6 | 0.067 | 37.6 ± 5.2 |
| **T92H** | -39.2 ± 7.9 | 6 | 0.41 | 60.5 ± 2.8 |
| **T92I** | -34.2 ± 8.9 | 5 | 0.69 | 57.6 ± 2.9 |
| **T92K** | -17.7 ± 5.2 | 5 | 0.30 | 50.6 ± 3.8 |
| **T92L** | -29.4 ± 9.4 | 5 | 0.98 | 52.5 ± 8.9 |
| **T92M** | -39.9 ± 9.6 | 5 | 0.43 | 45.6 ± 7.1 |
| **T92N** | -33.3 ± 8.3 | 6 | 0.73 | 11.8 ± 0.9 |
| **T92P** | -1.4 ± 0.8 | 5 | 0.032 | * |
| **T92Q** | -37.6 ± 9.0 | 6 | 0.52 | 56.8 ± 6.0 |
| **T92R** | -1.4 ± 0.4 | 5 | 0.032 | * |
| **T92S** | -28.3 ± 8.0 | 4 | 0.94 | 35.4 ± 4.0 |
| **T92V** | -45.7 ± 8.3 | 5 | 0.20 | 58.6 ± 4.3 |
| **T92W** | -30.2 ± 10.9 | 5 | 0.94 | 59.4 ± 4.9 |
| **T92Y** | -20.2 ± 5.9 | 4 | 0.42 | 61.3 ± 7.8 |

*not measured due to small current amplitudes (<2 pA/pF).
