# Supplementary material for: A pathogenic human Orai1 mutation unmasks STIM1-independent rapid inactivation of Orai1 channels
Source: eLife. 2023 Feb 20;12:e82281. doi: 10.7554/eLife.82281 (PMC9991058; doi:10.7554/eLife.82281)
Supplement: Figure 4—source data 1. [file elife-82281-fig4-data1.docx]

Figure 4 – Source Data. STIM1-independent inactivation of L138F and T92W Orai1.

**Figure 4C**

| **L138F Orai1 alone (1-I_ss_/I_peak_)** | | | | | |
| --- | --- | --- | --- | --- | --- |
| External Ca^2+^ | -120 mV | -100 mV | -80 mV | -60 mV | N |
| **20 mM Ca^2+^** | 0.57 ± 0.018 | 0.51 ± 0.023 | 0.44 ± 0.029 | 0.34 ± 0.028 | 8 |
| **Divalent-free** | 0.031 ± 0.004 | 0.031 ± 0.006 | 0.042 ± 0.007 | 0.048 ± 0.009 | 7 |

**Figure 4E**

| **T92W Orai1 alone (1-I_ss_/I_peak_)** | | | | | |
| --- | --- | --- | --- | --- | --- |
| External Ca^2+^ | -120 mV | -100 mV | -80 mV | -60 mV | N |
| **20 mM Ca^2+^** | 0.55 ± 0.039 | 0.52 ± 0.042 | 0.47 ± 0.045 | 0.43 ± 0.047 | 17 |
| **Divalent-free** | 0.13 ± 0.035 | 0.11 ± 0.035 | 0.09 ± 0.029 | 0.06 ± 0.025 | 5 |

| **T92W Orai1 alone (1-I_ss_/I_peak_)** | | | | | |
| --- | --- | --- | --- | --- | --- |
| External Ca^2+^ | -120 mV | -100 mV | -80 mV | -60 mV | N |
| **20 mM Ca^2+^** | 0.55 ± 0.039 | 0.52 ± 0.042 | 0.47 ± 0.045 | 0.43 ± 0.047 | 17 |
| **110 mM Ca^2+^** | 0.70 ± 0.040 | 0.69 ± 0.044 | 0.67 ± 0.045 | 0.68 ± 0.043 | 6 |

**Figure 4 – figure supplement 1A**

**Figure 4 – figure supplement 1C**

| **T92 Mutants % Inactivation** | | | |
| --- | --- | --- | --- |
| Mutant | Surface Area (Å^2^) | % Inactivation ± SEM | N |
| **T92V** | 155 | -72.7 ± 4.6 | 6 |
| **T92L** | 170 | 4.9 ± 1.1 | 5 |
| **T92I** | 175 | -12.8 ± 2.7 | 4 |
| **T92M** | 185 | 9.4 ± 1.3 | 5 |
| **T92H** | 195 | 12.1 ± 1.7 | 5 |
| **T92F** | 210 | 10.9 ± 0.8 | 5 |
| **T92Y** | 230 | 13.1 ± 1.5 | 6 |
| **T92W** | 255 | 31.5 ± 2.1 | 6 |

**Figure 4 – figure supplement 1E**

| **110 mM Ca^2+^ (1-I_ss_/I_peak_)** | | | | | |
| --- | --- | --- | --- | --- | --- |
| Mutant | -120 mV | -100 mV | -80 mV | -60 mV | N |
| **T92W** | 0.55 ± 0.039 | 0.52 ± 0.042 | 0.47 ± 0.045 | 0.43 ± 0.047 | 17 |
| **T92W/E106D** | 0.14 ± 0.053 | 0.14 ± 0.044 | 0.12 ± 0.048 | 0.15 ± 0.075 | 5 |
