# Supplementary material for: A pathogenic human Orai1 mutation unmasks STIM1-independent rapid inactivation of Orai1 channels
Source: eLife. 2023 Feb 20;12:e82281. doi: 10.7554/eLife.82281 (PMC9991058; doi:10.7554/eLife.82281)
Supplement: Figure 5—source data 1. [file elife-82281-fig5-data1.docx]

Figure 5 – Source Data. Dependence of T92W and WT Orai1 inactivation on local Ca^2+^.

**Figure 5C**

| **WT Orai1 + STIM1 (1-I_ss_/I_peak_)** | | | | | |
| --- | --- | --- | --- | --- | --- |
| Internal Solution | -120 mV | -100 mV | -80 mV | -60 mV | N |
| **0.8 mM BAPTA** | 0.39 ± 0.062 | 0.38 ± 0.056 | 0.29 ± 0.068 | 0.19 ± 0.093 | 4 |
| **8 mM BAPTA** | 0.25 ± 0.097 | 0.22 ± 0.062 | 0.14 ± 0.047 | 0.08 ± 0.040 | 4 |
| **20 mM BAPTA** | 0.20 ± 0.030 | 0.19 ± 0.028 | 0.13 ± 0.006 | 0.05 ± 0.014 | 6 |
| **10 mM EGTA** | 0.43 ± 0.029 | 0.35 ± 0.06 | 0.22 ± 0.024 | 0.09 ± 0.022 | 10 |
| **20 mM EGTA** | 0.43 ± 0.069 | 0.35 ± 0.081 | 0.22 ± 0.070 | 0.08 ± 0.044 | 4 |

**Figure 5D**

| **T92W Orai1 alone (1-I_ss_/I_peak_)** | | | | | |
| --- | --- | --- | --- | --- | --- |
| Internal Solution | -120 mV | -100 mV | -80 mV | -60 mV | N |
| **0.8 mM BAPTA** | 0.15 ± 0.074 | 0.12 ± 0.059 | 0.11 ± 0.045 | 0.09 ± 0.040 | 4 |
| **8 mM BAPTA** | 0.55 ± 0.039 | 0.52 ± 0.042 | 0.47 ± 0.045 | 0.43 ± 0.047 | 17 |
| **20 mM BAPTA** | 0.53 ± 0.058 | 0.50 ± 0.059 | 0.43 ± 0.069 | 0.39 ± 0.075 | 4 |
| **10 mM EGTA** | 0.11 ± 0.022 | 0.10 ± 0.017 | 0.09 ± 0.017 | 0.07 ± 0.016 | 9 |
| **20 mM EGTA** | 0.19 ± 0.041 | 0.16 ± 0.026 | 0.14 ± 0.022 | 0.11 ± 0.028 | 5 |
