# Supplementary material for: A pathogenic human Orai1 mutation unmasks STIM1-independent rapid inactivation of Orai1 channels
Source: eLife. 2023 Feb 20;12:e82281. doi: 10.7554/eLife.82281 (PMC9991058; doi:10.7554/eLife.82281)
Supplement: Figure 6—source data 1. [file elife-82281-fig6-data1.docx]

Figure 6 – Source Data. Paired pulse experiments reveal recovery of T92W inactivation through depolarizing steps.

**Figure 6A**

| **WT vs. T92W Orai1 (1-I_ss_/I_peak_)** | | | | | | |
| --- | --- | --- | --- | --- | --- | --- |
| Mutant | Internal Solution | -120 mV | -100 mV | -80 mV | -60 mV | N |
| **WT + STIM1** | **10 mM EGTA** | 0.43 ± 0.029 | 0.35 ± 0.06 | 0.22 ± 0.024 | 0.09 ± 0.022 | 10 |
| **T92W alone** | **8 mM BAPTA** | 0.55 ± 0.039 | 0.52 ± 0.042 | 0.47 ± 0.045 | 0.43 ± 0.047 | 17 |

**Figure 6D**

| **WT + STIM1 Orai1 (1-I_ss_/I_peak_)** | | | | |
| --- | --- | --- | --- | --- |
| External Ca^2+^ | 1^st^ Pulse | 2^nd^ Pulse | Paired T-test  p-value | N |
| **2 mM Ca^2+^** | 0.19 ± 0.092 | 0.22 ± 0.072 | 0.78 | 6 |
| **20 mM Ca^2+^** | 0.31 ± 0.023 | 0.34 ± 0.024 | 0.055 | 7 |
| **110 mM Ca^2+^** | 0.40 ± 0.026 | 0.46 ± 0.025 | 0.027 | 6 |

**Figure 6F**

| **T92W Orai1 alone (1-I_ss_/I_peak_)** | | | | |
| --- | --- | --- | --- | --- |
| External Ca^2+^ | 1^st^ Pulse | 2^nd^ Pulse | Paired T-test  p-value | N |
| **2 mM Ca^2+^** | 0.18 ± 0.028 | 0.31 ± 0.046 | 0.011 | 8 |
| **20 mM Ca^2+^** | 0.14 ± 0.019 | 0.20 ± 0.027 | 2.2*10^-3^ | 7 |
| **110 mM Ca^2+^** | 0.10 ± 0.033 | 0.17 ± 0.043 | 8.1*10^-4^ | 8 |

**Figure 6 – figure supplement 1B**

| **T92W Orai1 8 mM BAPTA (1-I_ss_/I_peak_)** | | | | |
| --- | --- | --- | --- | --- |
| External Ca^2+^ | 1^st^ Pulse | 2^nd^ Pulse | Paired T-test  p-value | N |
| **2 mM Ca^2+^** | 0.48 ± 0.063 | 0.53 ± 0.075 | 0.043 | 4 |
| **20 mM Ca^2+^** | 0.50 ± 0.039 | 0.56 ± 0.040 | 3.5*10^-3^ | 6 |
| **110 mM Ca^2+^** | 0.55 ± 0.036 | 0.69 ± 0.033 | 8.3*10^-6^ | 6 |

**Figure 6 – figure supplement 1D**

| **L138F Orai1 10 mM EGTA (1-I_ss_/I_peak_)** | | | | |
| --- | --- | --- | --- | --- |
| External Ca^2+^ | 1^st^ Pulse | 2^nd^ Pulse | Paired T-test  p-value | N |
| **2 mM Ca^2+^** | 0.22 ± 0.027 | 0.24 ± 0.023 | 0.28 | 5 |
| **20 mM Ca^2+^** | 0.30 ± 0.038 | 0.32 ± 0.032 | 0.079 | 6 |
| **110 mM Ca^2+^** | 0.26 ± 0.056 | 0.32 ± 0.058 | 4.9*10^-3^ | 6 |
