# Supplementary material for: A pathogenic human Orai1 mutation unmasks STIM1-independent rapid inactivation of Orai1 channels
Source: eLife. 2023 Feb 20;12:e82281. doi: 10.7554/eLife.82281 (PMC9991058; doi:10.7554/eLife.82281)
Supplement: Figure 7—source data 1. [file elife-82281-fig7-data1.docx]

Figure 7 – Source Data. Effects of N- and C-terminal mutations on T92W CDI.

**Figure 7C**

| **T92W C-terminal deletion (1-I_ss_/I_peak_)** | | | | | |
| --- | --- | --- | --- | --- | --- |
| Mutant | -120 mV | -100 mV | -80 mV | -60 mV | N |
| **T92W** | 0.55 ± 0.039 | 0.52 ± 0.042 | 0.47 ± 0.045 | 0.43 ± 0.047 | 17 |
| **T92W del267-301** | 0.28 ± 0.047 | 0.27 ± 0.047 | 0.24 ± 0.054 | 0.19 ± 0.048 | 5 |

**Figure 7F**

| **T92W N-terminal Mutants (1-I_ss_/I_peak_)** | | | | | |
| --- | --- | --- | --- | --- | --- |
| Mutant | -120 mV | -100 mV | -80 mV | -60 mV | N |
| **T92W** | 0.55 ± 0.039 | 0.52 ± 0.042 | 0.47 ± 0.045 | 0.43 ± 0.047 | 17 |
| **W76E/T92W** | 0.79 ± 0.053 | 0.79 ± 0.047 | 0.76 ± 0.038 | 0.71 ± 0.037 | 4 |
| **Y80A/T92W** | 0.80 ± 0.017 | 0.76 ± 0.017 | 0.72 ± 0.040 | 0.66 ± 0.059 | 6 |
| **Y80E/T92W** | 0.86 ± 0.018 | 0.83 ± 0.026 | 0.75 ± 0.048 | 0.73 ± 0.044 | 4 |
| **R83E/T92W** | 0.78 ± 0.025 | 0.70 ± 0.014 | 0.63 ± 0.022 | 0.58 ± 0.024 | 4 |

| **Orai1 alone** | | | |
| --- | --- | --- | --- |
| Mutant | Current Density (pA/pF ± SEM) | N | T-test p-value  (versus T92W) |
| **T92W** | -27.7 ± 3.8 | 8 | N/A |
| **del2-85 T92W** | -0.7 ± 0.2 | 6 | 1.9*10^-4^ |
| **K85E/T92W** | -0.7 ± 0.1 | 5 | 1.9*10^-4^ |

**Figure 7 – figure supplement 1C**
