# Supplementary material for: A pathogenic human Orai1 mutation unmasks STIM1-independent rapid inactivation of Orai1 channels
Source: eLife. 2023 Feb 20;12:e82281. doi: 10.7554/eLife.82281 (PMC9991058; doi:10.7554/eLife.82281)
Supplement: Figure 8—source data 1. [file elife-82281-fig8-data1.docx]

Figure 8 – Source Data. STIM1 modulates the Ca^2+^ sensitivity of T92W CDI.

**Figure 8D**

| **10 mM EGTA (1-I_ss_/I_peak_)** | | | | | |
| --- | --- | --- | --- | --- | --- |
| Mutant | -120 mV | -100 mV | -80 mV | -60 mV | N |
| **WT + STIM1** | 0.43 ± 0.029 | 0.35 ± 0.06 | 0.22 ± 0.024 | 0.09 ± 0.022 | 10 |
| **T92W** | 0.11 ± 0.022 | 0.10 ± 0.017 | 0.09 ± 0.017 | 0.07 ± 0.016 | 9 |
| **T92W + STIM1** | 0.39 ± 0.085 | 0.32 ± 0.075 | 0.22 ± 0.061 | 0.12 ± 0.040 | 5 |

**Figure 8E**

| **8 mM BAPTA (1-I_ss_/I_peak_)** | | | | | |
| --- | --- | --- | --- | --- | --- |
| Mutant | -120 mV | -100 mV | -80 mV | -60 mV | N |
| **WT + STIM1** | 0.25 ± 0.097 | 0.22 ± 0.062 | 0.14 ± 0.047 | 0.08 ± 0.040 | 4 |
| **T92W** | 0.55 ± 0.039 | 0.52 ± 0.042 | 0.47 ± 0.045 | 0.43 ± 0.047 | 17 |
| **T92W + STIM1** | 0.20 ± 0.042 | 0.20 ± 0.038 | 0.17 ± 0.033 | 0.12 ± 0.027 | 5 |

**Figure 8 – figure supplement 1B**

| **Y80E/T92W + STIM1 (1-I_ss_/I_peak_)** | | | | | |
| --- | --- | --- | --- | --- | --- |
| Mutant | -120 mV | -100 mV | -80 mV | -60 mV | N |
| **Y80E/T92W alone** | 0.86 ± 0.018 | 0.83 ± 0.026 | 0.75 ± 0.048 | 0.73 ± 0.044 | 4 |
| **Y80E/T92W + STIM1** | 0.25 ± 0.069 | 0.31 ± 0.091 | 0.29 ± 0.091 | 0.31 ± 0.14 | 4 |
